# Notes on a Case of Splenomegalic Biliary Cirrhosis, in a Boy Aged Six Years
1Read at a meeting of the Society on January 10th, 1906.


**Published:** 1906-03

**Authors:** Edward Cecil Williams

**Affiliations:** Physician to the Royal Hospital for Sick Children and Women, Bristol


### NOTES ON A CASE OF SPLENOMEGALIC BILIARY CIRRHOSIS, IN A BOY AGED SIX YEARS.[1]

BY

EDWARD CECIL WILLIAMS, M.B. Cantab.,

*Physician to the Royal Hospital for Sick Children and Women, Bristol.*

---

THE notes of this case are of interest, as the number of recorded cases in this country is few, the pathology of the disease is as yet uncertain, as is also the exact rôle played by syphilis, when a history of that disease is obtainable. Then there is the fact that in many of these cases there is an arrest of growth.

The child had always been delicate; two and a half years ago had jaundice and was said to have an enlarged liver, has been yellow ever since. Appetite has always been good. Never had any sickness or diarrhœa. Bowels are regular,

[1] Read at a meeting of the Society on January 10th, 1906.

motions inclined to be white, urine never been dark brown. Never had any pain except in left side occasionally. Mother died of consumption, brothers and sisters said to be healthy. Has suffered from shortness of breath. Legs never swell. A short time ago had blue patches the size of a shilling on legs and abdomen, which were tender.

On admission he was pale, conjunctivæ slightly yellow, skin of body and limbs of a lemon tint, enlarged veins over chest and abdomen. Heart's apex beat is in fifth space in nipple line, no increase in cardiac dulness, hæmic murmur at base, other sounds normal. Lungs appear healthy. Abdomen very distended, girth 26 in., distended surface veins. Liver much enlarged, dulness extending from the sixth rib to the level of the umbilicus, where its free edge can be felt; left lobe of liver can be felt below the ensiform cartilage. Spleen also much enlarged, extending in the mid-axillary line within a couple of finger-breadths of the iliac crest; its notch can be felt on its outer border, about 2½ to 3 inches from the umbilicus; it is dense and smooth. There is no evidence of ascites, no œdema of legs, but on each leg are a few petechiæ. Urine acid, 1027, no albumin or sugar, trace of bile (Gmelin's test). No enlargement of lymphatic glands. There is marked clubbing of fingers and toes. Height, 3 ft. 4 in.; weight, 36½ lbs.

Blood: hæmoglobin, 62 per cent.; red cells, 5,400,000; white cells, 9,545 per c. mm. Differential count: polynuclear, 38.1 per cent.; uninuclear, 3.3 per cent.; eosinophile, 11.6 per cent.; lymphocytes, 46 per cent.; basophile, 1 per cent.

There is nothing distinctive in the blood picture. On admission his temperature was practically normal until October 12th, when there was a rise of temperature over 101° F., with an increase of jaundice. With the fall of temperature there has gradually been a diminution of the jaundice; now the only trace remains in a slight conjunctival tinge. The motions now are yellow. There has also been, coincident in some degree with the disappearance of jaundice, a gradual diminution of the abdominal girth by 2½ inches. The father thinks the boy's growth during the last 2½ years has been inappreciable. Gilbert and Fournier have described a juvenile form of this disease characterised by great splenomegaly, of which this case is an example. The clubbing of fingers is also a more constant symptom of the disease than in the adult form. The clubbing is not due to any bony increase, but to hyperplasia of the soft tissues, probably the result of the irritation of toxins. Whether the hepatic or splenic enlargement be primary cannot be ascertained. It is probable the enlargement of both organs may be due to the circulation in the blood of some toxin, which may act selectively more especially on the spleen, and then passing along to the liver with the blood, may be excreted by the small bile-ducts, causing a descending cholangitis; or it may be due to some poison manufactured in the intestine ascending to the smaller bile-ducts, and so to the spleen along the splenic vein. Syphilis does not appear to play a direct part in these cases; there is a doubtful history of paternal syphilis in this case, but none of the usual symptoms of congenital syphilis. Jaundice is never very deep, in fact cases may occur without any jaundice at all. This condition cannot have anything to do with Banti's disease, which is the termination of the splenic anæmia of adults with multilobular cirrhosis and ascites. The second dentition is the earliest date at which the splenic anæmia of adults occurs, and the splenic anæmia of infants is a totally distinct affection, which seldom occurs after the age of two years. At the present time my patient seems to be improving; whether this improvement will result in cure only time can show. When these cases tend to go downhill there is a tendency to ascites, œdema of legs, and albuminuria, with the mixed type of cirrhosis.

Treatment consists in keeping the patient warm and suitably clad. Diet should be plain, and bowels kept well open. As drugs calomel, gr. $\frac{1}{20}$ three times daily, and salicylate of soda, as intestinal disinfectants, seem to answer best. I have not seen any good from active anti-syphilitic treatment. The surgical treatment of draining the gall-bladder is based on the assumption of an ascending cholangitis, but until there is more evidence on this point it cannot be regarded as an established procedure.

Photographs of this case have also been exhibited before the Society for the Study of Disease in Children.

### REFERENCES.

**Gilbert and Fournier**—*Rev. mens. d. Mal. de l'Enf.*, 1895.
**F. Taylor**—*Guy's Hosp. Rep.*, 1900 (1897), liv. 1.
**Wilson**—*Tr. Clin. Soc. Lond.*, 1890, xxiii. 162.
**Macalister**—*Rep. Soc. Study Dis. Child.*, 1902, i. 199.
**Sir T. Barlow and H. B. Shaw**—*Tr. Clin. Soc. Lond.*, 1902, xxxv. 155.
**F. Taylor**—Lumleian Lecture, *Lancet*, 1904, i. 1477 *et seq.*
**Parkes Weber**—*Rep. Soc. Study Dis. Child.*, 1905, v.

